# Unveiling the Female Factor: Gender-Based Differences in Outcomes and Survival Following Radical Cystectomy for Bladder Cancer

**DOI:** 10.3390/cancers18020308

**Published:** 2026-01-20

**Authors:** Federico Ceria, Gad Muhammad, Francesco Del Giudice, Youssef Ibrahim, Ramesh Thurairaja, Rajesh Nair, Elsie Mensah, Muhammad Shamim Khan, Yasmin Abu Ghanem

**Affiliations:** 1 Guy’s and St. Thomas’ NHS Foundation Trust, Guy’s Hospital, London SE1 7EH, UK; f.ceria@studenti.unisr.it (F.C.); rajesh.nair10@nhs.net (R.N.); elsie.mensah@gstt.nhs.uk (E.M.); shamim.khan10@nhs.net (M.S.K.); 2 Department of Maternal-Infant and Urological Sciences, “Sapienza” University of Rome, Umberto I Hospital, 00185 Rome, Italy; 3 Department of Urology, Stanford University School of Medicine, Stanford, CA 94304, USA

**Keywords:** gender, cystectomy, survival

## Abstract

Background: Female patients undergoing radical cystectomy (RC) for bladder cancer have historically presented with more advanced disease and poorer survival outcomes than males. These disparities have been attributed to biological differences, delayed diagnosis, and variations in treatment delivery. Recent data suggest, however, that outcomes may converge when patients are managed in standardized, multidisciplinary, high-volume centers. This study evaluated the influence of gender on perioperative features and oncological outcomes such as disease-free survival (DFS), disease-specific survival (DSS), and overall survival (OS) at a tertiary uro-oncology center. Methods: We retrospectively reviewed a prospectively maintained database of patients who underwent open or robotic-assisted RC for histologically confirmed urothelial carcinoma between 2014 and 2023 at Guy’s and St. Thomas’ NHS Foundation Trust. Demographic, perioperative, and pathological variables were stratified by gender to assess their association with DFS, DSS, and OS. Results: A total of 887 patients were included: 640 men (72.2%) and 247 women (27.8%), with similar mean age (68.5 vs. 68.1 years) and tumour histology (pure urothelial carcinoma 85% vs. 83%). Men had a higher prevalence of diabetes and chronic kidney disease, but no significant differences were observed in time from referral to surgery (0.93 vs. 1.03 months, *p* = 0.93), use of neoadjuvant therapy (21.6% vs. 17.3%, *p* = 0.25), or surgical approach (*p* = 0.55). Pathological stage distribution was comparable between sexes (pT0–1: 44% vs. 50%; pT2–4: 56% vs. 50%; *p* = 0.13). Kaplan–Meier analysis revealed no significant gender-related differences in 12-month DFS (77.3% vs. 75.4%, *p* = 0.20), DSS (85.6% vs. 86.9%, *p* = 0.56), or OS (81.2% vs. 85.2%, *p* = 0.70). Conclusion: In this high-volume tertiary center, gender did not independently influence perioperative or survival outcomes following radical cystectomy. These findings suggest that standardized, multidisciplinary management within specialized bladder cancer pathways may mitigate the pathological and survival disparities historically associated with gender.

## Simple Summary

This study looked at whether men and women with bladder cancer have different outcomes after their bladder is surgically removed in a specialised cancer centre. Although women with bladder cancer are often thought to have more advanced disease and worse survival, this is not always due to the cancer itself and may be influenced by delays in diagnosis or differences in access to specialist care. In this large single-centre study, men and women received similar investigations, treatments, and types of surgery and had comparable tumour features at the time of operation. The results showed no meaningful differences between men and women in cancer control or survival after surgery, suggesting that when both sexes receive timely, standardised care in a high-volume expert centre, long-standing survival gaps between men and women can be greatly reduced.

## 1. Introduction

Bladder cancer (BC) is the tenth most common malignancy worldwide and the seventh most frequent among men [1,2,3,4,5]. Although men are diagnosed more often, with a male-to-female incidence ratio of roughly 4:1, women usually present with more advanced disease and experience poorer outcomes, including higher rates of recurrence, progression, and cancer-specific mortality [1,2,3,4,5,6,7]. Several studies have linked female gender to advanced tumour stage and lower survival following both open and robotic radical cystectomy for urothelial and variant histologies [2,3,4,5,6,7,8].

Despite consistent evidence of a survival gap between men and women, it remains uncertain whether treatment within tertiary referral centres can mitigate these sex-based differences in outcome [2,3,4,6,7,8]. Several factors may contribute to this disparity [2,3,4,5,6,7,9,10]. Women often underrecognize haematuria, the most common presenting symptom of BC, and are more frequently affected by urinary tract infections, particularly under the age of 40, which can mimic BC symptoms and delay diagnosis [7,9,10,11]. These diagnostic delays, including delayed imaging, specialist referral, and treatment initiation, may contribute to poorer outcomes in women [2,3,4,7,9,10,11].

Anatomical and physiological differences may also influence tumour behaviour and surgical outcomes [3,4,6,7,8,12]. Men are more prone to bladder outlet obstruction and detrusor hypertrophy secondary to prostate enlargement [3,4,12]. In contrast, women have a thinner bladder wall, which may predispose them to non-organ-confined disease and higher postoperative complication rates [3,4,6,7,8,12]. In addition, the prostatic capsule may serve as a physical barrier to tumour spread in men, while in women, the periurethral tissue is more susceptible to extravesical extension [3,4,6,7,8,12].

Molecular and embryological factors further shape gender differences in BC [2,3,4,6,7,8,13,14,15,16,17]. Urothelial carcinoma shows sex-specific expression of androgen and estrogen receptors; notably, female urothelium has higher estrogen receptor levels, particularly in the trigone and posterior neck, which has been associated with more advanced disease stage and grade [2,3,6,8,13,14,15,16]. The shared embryologic origin of the bladder trigone and vagina, along with differences in perivesical lymphatic anatomy, may facilitate extravesical spread in women [2,3,13,17]. Socioeconomic and environmental influences such as smoking, occupational exposure, and disparities in healthcare access also play a role in sex-related variations in BC outcomes [1,4,5,7,10,11,18].

Beyond these well-established factors, emerging evidence suggests that tumour genomics, immune microenvironment, and the urinary microbiome contribute to gender-specific patterns of disease progression. However, their clinical implications remain incompletely understood [13,14,16,19,20,21].

Sex differences extend to surgical management as well. Women undergoing cystectomy are less likely to receive orthotopic neobladder reconstruction, often due to concerns about urethral recurrence, with ileal conduit diversion being the most frequently selected option [22,23,24,25]. Moreover, perioperative variables such as operative time, blood loss, and complication rates may vary between sexes and influence postoperative outcomes [3,4,5,6,8,12,23]. In this context, the present study examines a large, single-centre cohort of patients who underwent radical cystectomy for bladder cancer. We compare preoperative, intraoperative, and pathological features between men and women and assess survival outcomes by gender. The aim is to evaluate whether management in a tertiary referral centre can help narrow the gender gap in bladder cancer survival.

## 2. Materials and Methods

### 2.1. Study Design and Population

A retrospective analysis was performed using a prospectively maintained institutional database. All patients who underwent radical cystectomy (RC) for urothelial carcinoma of the bladder between 2014 and 2023 were identified and included. Patients with non-urothelial histology or RC performed for other oncologic or benign indications were excluded. The study population was stratified according to biological sex (male and female).

### 2.2. Assessed Variables

Preoperative variables compared between the two cohorts included age at diagnosis and at cystectomy, ethnicity, body mass index (BMI), and comorbidities such as diabetes mellitus (DM) and chronic kidney disease (CKD). Laboratory parameters included estimated glomerular filtration rate (eGFR) and haemoglobin (Hb) level. The presence of carcinoma in situ (CIS) and receipt of neoadjuvant therapy were also documented.

Surgical variables comprised operative approach (open vs. robotic-assisted), operative time, estimated blood loss (EBL), and number of lymph nodes retrieved. Postoperative and pathological characteristics included pathological T stage, histological subtype, presence of concomitant CIS, and surgical margin status (positive surgical margins- PSM).

### 2.3. Statistical Analysis

Perioperative and pathological variables were analyzed for associations with survival outcomes within each sex-specific cohort. Multivariate models were developed to identify perioperative factors independently associated with differences in survival between men and women. Kaplan–Meier methods were used to estimate disease-free survival (DFS), disease-specific survival (DSS), and overall survival (OS), and the log-rank test was applied for group comparisons.

Continuous variables were summarized as medians with interquartile ranges (IQR), and categorical variables as frequencies and percentages. Between-group differences were assessed using the Chi-square or Fisher’s exact test for categorical variables, and the Mann–Whitney U test for continuous variables due to non-normal distributions. Ninety-five percent confidence intervals (95% CI) were calculated for median DFS, DSS, and OS. All *p*-values were two-tailed, with statistical significance defined as *p* < 0.05. Statistical analyses were performed using R software, version 4.5.1 (R Core Team, 13 June 2025).

### 2.4. Diagnosis, Treatment and Pathological Evaluation

At initial diagnosis, all patients underwent bimanual examination under anesthesia immediately before transurethral resection of the bladder tumour (TURBT). Preoperative evaluation included abdominal and pelvic computed tomography (CT) and chest radiography. All TURBT and RC specimens were reviewed by dedicated genitourinary pathologists. For patients referred from external institutions, original slides were re-evaluated, and when necessary, repeat TURBT was performed at our center.

Radical cystectomy was indicated for muscle-invasive bladder cancer (MIBC) or high-risk non-muscle-invasive bladder cancer (NMIBC) refractory to intravesical therapy. The procedure included removal of the bladder and distal ureters, together with the seminal vesicles and prostate in male patients, and the uterus, ovaries, and anterior vaginal wall in female patients. A limited pelvic lymph node dissection (PLND) was routinely performed.

Postoperative follow-up consisted of clinical and radiologic assessment every three months during the first year, every six months until year five, and annually thereafter, at the discretion of the treating physician. Follow-up duration was calculated from the date of RC to recurrence, death, or last documented visit.

## 3. Results

### 3.1. Patient Population

Of 1002 patients entered in the institutional database, 884 who underwent radical cystectomy (RC) for urothelial carcinoma of the bladder between 2014 and 2023 met the inclusion criteria and were analyzed. The remaining patients were excluded because their procedures were performed for non-malignant or alternative indications, including radiation-induced bladder dysfunction, refractory benign disease, or functional abnormalities.

The final study cohort comprised 639 men and 245 women. Median age at surgery was 69.8 years (IQR 13.3) in men and 70.5 years (IQR 12.6) in women. Median body mass index (BMI) was 27.1 (IQR 6.48) and 26.7 (IQR 6.58), respectively.

Diabetes mellitus (DM) was present in 75 men (11.7%) and 26 women (10.6%), while chronic kidney disease (CKD) was documented in 105 men (16.4%) and 33 women (13.5%). Median estimated glomerular filtration rate (eGFR) was comparable between sexes 71 mL/min/1.73 m^2^ (IQR 28) in men and 73 mL/min/1.73 m^2^ (IQR 25) in women. Neoadjuvant systemic therapy was administered in 105 men (16.4%) and 33 women (13.5%) (*p* = 0.21), with no difference in treatment allocation. The median interval from referral to RC was 0.93 months for men and 1.03 months for women (*p* = 0.93).

Clinical stage at diagnosis and the presence of concomitant carcinoma in situ (CIS) were similar across sexes, indicating comparable baseline disease characteristics. Overall, preoperative demographic, clinical, and pathological variables were well balanced between groups, with no statistically significant differences. A detailed summary of baseline characteristics is presented in Table 1.

### 3.2. Surgical Characteristics

Open radical cystectomy (ORC) was performed in 194 men (30.4%) and 68 women (27.8%), whereas robot-assisted radical cystectomy (RARC) was undertaken in 429 men (67.1%) and 172 women (70.2%) (*p* = 0.47). Median operative time was 330 min for both sexes (IQR 120 in men; IQR 90 in women). Median estimated blood loss (EBL) was 400 mL in both cohorts (IQR 9 for men; IQR 12 for women). The median number of lymph nodes retrieved was 15 (IQR 9) in men and 16 (IQR 12) in women.

No statistically significant sex-based differences were identified for intraoperative parameters. Surgical characteristics are summarised in Table 2.

### 3.3. Pathological Findings

Pathological assessment included tumour stage, histological subtype, surgical margin status, and ureteral or urethral involvement. Positive surgical margins (PSM) were found in 41 men (6.4%) and 15 women (6.1%), with a similar distribution of ureteric and urethral involvement across groups. The distribution of pathological T stage and the frequency of variant histology were also comparable between sexes. Comprehensive pathological data are provided in Table 3.

### 3.4. Survival Outcomes

**Figure 1:**
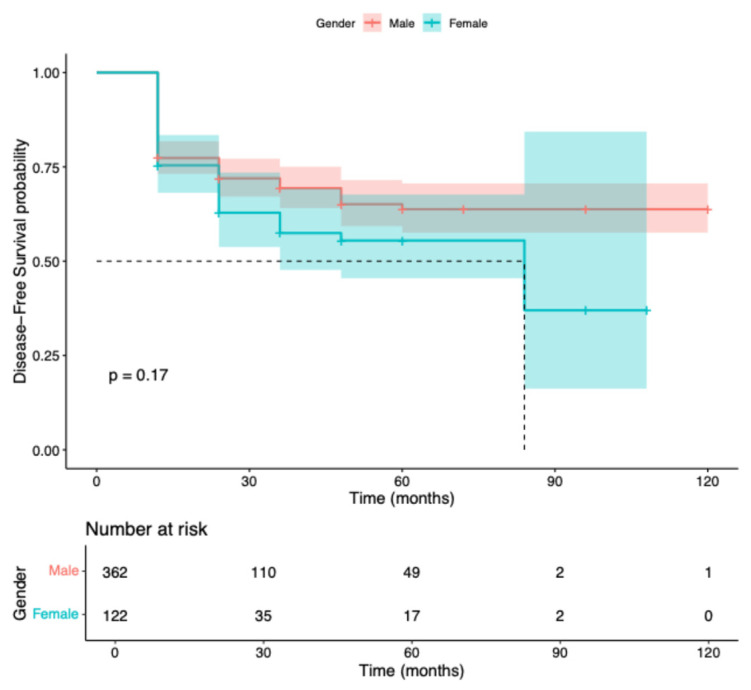
Kaplan–Meier curves for disease-free survival (DFS) stratified by gender (Male vs. Female) in the analysis group. Median follow-up was measured in months. The log-rank test showed no statistically significant difference between groups (*p* = 0.17). Numbers at risk are reported below the curves at selected time points.

Of the 639 men and 245 women included, 362 men and 122 women had complete follow-up data and were evaluable for disease-free survival (DFS). Tumour recurrence occurred in 104 men and 44 women. The estimated 12-month DFS was 77.3% (95% CI 73.2–81.8) in men and 75.4% (95% CI 68.1–83.5) in women, with no statistically significant difference between groups (log-rank test, χ^2^ = 1.9, *p* = 0.17). Estimated 5-year DFS was 60% in men and 52% in women (Figure 1).

**Figure 2:**
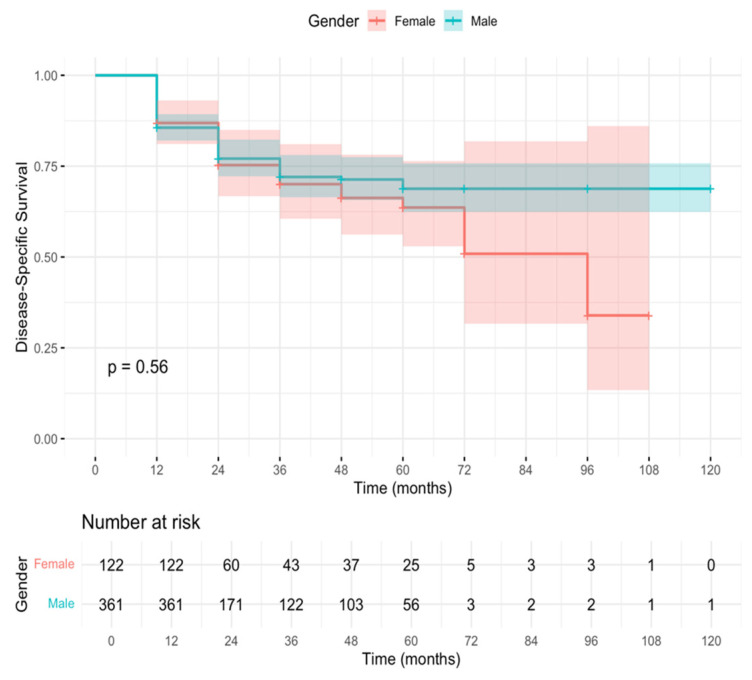
Kaplan–Meier curves for disease-specific survival (DSS) stratified by gender (Male vs. Female) in the analysis group. Median follow-up was measured in months. The log-rank test showed no statistically significant difference between groups (*p* = 0.56). Numbers at risk are reported below the curves at selected time points.

For disease-specific survival (DSS), 361 men and 122 women were included. The 12-month DSS rates were 85.6% (95% CI 82.1–89.3) in men and 86.9% (95% CI 81.1–93.1) in women. The 5-year DSS was 68% in men and 62% in women, without a statistically significant difference (log-rank test, χ^2^ = 0.26, *p* = 0.56) (Figure 2).

**Figure 3:**
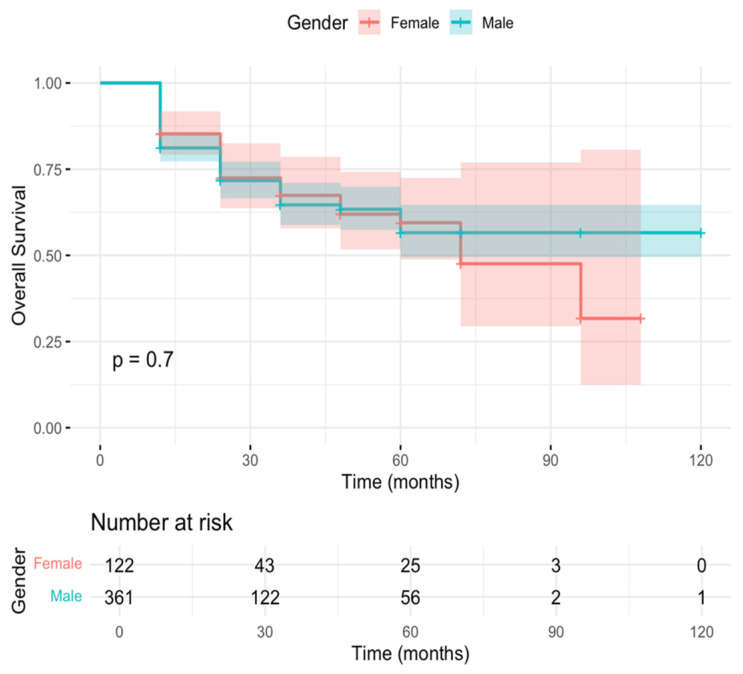
Kaplan–Meier curves for overall survival (OS) stratified by gender (Male vs. Female) in the analysis group. Median follow-up was measured in months. The log-rank test showed no statistically significant difference between groups (*p* = 0.7). Numbers at risk are reported below the curves at selected time points.

Similarly, 361 men and 122 women were included in the overall survival (OS) analysis. Estimated 12-month OS rates were 81.2% (95% CI 77.2–85.3) for men and 85.2% (95% CI 79.2–91.8) for women (Figure 3). No significant difference in OS was observed between sexes (log-rank test, χ^2^ = 0.1, *p* = 0.7).

## 4. Discussion

In the current study, we analysed 884 patients who underwent radical cystectomy (RC) for urothelial carcinoma of the bladder. No significant sex-based differences were found in perioperative, surgical, or pathological characteristics, nor in disease-free, disease-specific, or overall survival. These findings suggest that within a high-volume tertiary referral centre with standardised bladder cancer pathways, the long-recognised outcome disparity between men and women may be substantially reduced.

Previous studies have repeatedly shown that women with bladder cancer tend to present with more advanced disease and have poorer cancer-specific and overall survival than men [2,3,4,5,6,7,8,13,24]. Several explanations have been proposed, including diagnostic delay, anatomical differences, and unequal access to treatment. However, they have been described primarily in population-based settings, where variability in expertise, referral timing, and management is common. Our findings indicate that when both sexes receive timely, guideline-concordant care within a centralised multidisciplinary framework, these historical differences can largely disappear.

Notably, women in our cohort received neoadjuvant chemotherapy at rates comparable to men, which is consistent with guideline recommendations but contrasts with population-based data showing that women are less likely to receive NAC despite similar eligibility [26,27]. This treatment parity may have contributed to the equitable outcomes observed, as NAC has been shown to improve pathological downstaging and survival in both sexes when appropriately administered [1,26].

In our cohort, the interval from referral to surgery was virtually identical between men and women (0.93 vs. 1.03 months, *p* = 0.93), suggesting equivalent diagnostic and treatment access. This contrasts with previous registry-based analyses reporting longer time-to-treatment intervals among women, which have been associated with worse outcomes [2,9,10]. Similarly, intraoperative and perioperative parameters, surgical approach, operative time, blood loss, and lymph node yield were comparable between sexes, whereas earlier reports described greater morbidity in women [3,6,8,24]. The median lymph node yield in our cohort was similar as well, with 15 for men and 16 for women (*p* = 0.21). Adequate lymphadenectomy is a critical quality metric associated with improved staging accuracy and survival [28,29], and the consistency in our results suggests that surgical quality was maintained regardless of patient sex. Previous studies have reported variability in the extent of lymph node dissection performed in women, which may contribute to outcome disparities in less standardised settings [28].

Pathological findings were also balanced, with no significant differences in pathological T stage, histological variants, concomitant carcinoma in situ, or positive surgical margins. Earlier studies linked female sex to a higher likelihood of non-organ-confined disease [2,3,6,13,24]. The homogeneity in our results may reflect the impact of standardised surgical protocols, centralised pathology review, and consistent adherence to oncologic quality indicators.

Postoperative complications occurred at similar rates between sexes in our cohort (Clavien–Dindo ≥ 3: 9.1% men vs 10.9% women, *p* = 0.07). This finding contrasts with previous reports suggesting that women experience higher complication rates, particularly pelvic organ prolapse and voiding dysfunction after neobladder reconstruction [30,31]. The comparable safety profile observed may reflect refined surgical techniques, including nerve-sparing and organ-sparing approaches when oncologically feasible, as well as standardized perioperative care protocols [1,30].

The absence of survival disparities is particularly noteworthy. The 12-month DFS was 77.3% for men and 75.4% for women (*p* = 0.17); DSS was 85.6% versus 86.9% (*p* = 0.56); and OS was 81.2% versus 85.2% (*p* = 0.70). Median DFS and OS were reached only among women (84 and 72 months, respectively), whereas they were not reached among men, reflecting low event rates and extended follow-up. Importantly, no early survival disadvantage was observed in women, contrasting with prior reports showing the highest excess mortality within the first two years after diagnosis [4,7]. These results support the hypothesis that system-level factors, rather than intrinsic biological differences alone, drive much of the sex-based survival gap observed in broader populations.

Beyond healthcare organisation, our findings may also suggest that when clinical and pathological variables are balanced, biological variability between sexes exerts a smaller influence on outcomes. Although molecular and genomic data were not available, previous research has implicated androgen and estrogen receptor signalling, immune microenvironmental characteristics, and the urinary microbiome in sex-specific tumour behaviour [13,14,15,16,17,19,20,21,32]. The role of sex hormones in bladder cancer biology remains an active area of investigation. Estrogen receptor-β expression is higher in female bladder tissue, particularly in the trigone, and has been associated with more aggressive tumour phenotypes [15,16,17]. Conversely, androgen receptor expression appears inversely correlated with tumour stage and may confer protective effects [15]. Whether hormonal manipulation could improve outcomes in selected patients warrants further study [33,34]. In our cohort, the absence of outcome differences despite these biological distinctions suggests that contemporary multimodal therapy may overcome hormonal influences on tumour behaviour. Future studies integrating molecular profiling could determine whether the equalisation of outcomes in specialised centres corresponds to convergence at the biological level.

The choice of urinary diversion may also influence postoperative outcomes and quality of life. Although overall neobladder utilization was relatively low in our cohort (3.2% women vs 5.6% men, *p* = 0.4), reflecting contemporary patient selection and institutional practice patterns, the absence of sex-based disparity in access to continent diversion contrasts with registry data showing that women are less likely to receive this option despite meeting selection criteria [25,35]. Equal access to reconstructive options in our centre may have contributed to comparable functional outcomes and patient satisfaction between sexes, although formal quality-of-life assessment was not performed in this study [35,36].

This study has several limitations. The retrospective, single-centre design introduces potential selection bias and limits generalizability compared with multicentre or registry-based studies. We acknowledge that molecular data were unavailable, which limited our ability to explore hormonal or immune mechanisms that might underlie sex differences. However, since this study was carried out in a high-volume tertiary centre with well-established care pathways, our results likely reflect optimised outcomes and may not be directly comparable to those from smaller or non-specialist hospitals.

Overall, our findings indicate that the organisation and delivery of care are as critical as biological factors in determining sex equity in bladder cancer outcomes. Centralised, multidisciplinary management and equitable access to neoadjuvant chemotherapy and expert surgery appear capable of neutralising long-standing sex-based survival differences. The benefits of high-volume centralisation extend beyond technical expertise. Specialised bladder cancer units facilitate multidisciplinary tumour board discussions, access to clinical trials, and comprehensive supportive care, all of which may reduce sex-based disparities [37,38]. Evidence from national audits demonstrates that hospital volume correlates with improved outcomes after radical cystectomy, with the greatest survival gains observed in women treated at high-volume centres [37]. Our results support policy initiatives promoting centralisation of complex cancer surgery to improve equity of care.

From a practical perspective, these data support continued centralisation of radical cystectomy and bladder cancer care within high-volume, guideline-driven centres. Ensuring prompt referral, consistent access to systemic therapy, and standardised perioperative pathways may represent the most effective strategy to close the sex-related survival gap observed in population-based cohorts. At the same time, future research should integrate molecular and genomic analyses to determine whether equalisation of clinical outcomes is accompanied by convergence at the biological level. Such studies could clarify whether residual variability between sexes reflects subtle molecular differences or immune and hormonal interactions that become clinically silent in optimised care environments.

## 5. Conclusions

In summary, men and women undergoing radical cystectomy for urothelial bladder cancer demonstrated comparable perioperative, pathological, and survival outcomes when treated within a high-volume tertiary referral centre. Our results indicate that the survival difference between men and women is not fixed. When both sexes receive timely and consistent care in experienced centres, outcomes become comparable. In practice, improving access to specialist teams and standardising treatment pathways may be the most effective way to close this gap.

Future prospective, multicenter studies ideally incorporating molecular and genomic profiling are warranted to determine whether any residual biological differences persist once healthcare delivery factors are optimised.

## Figures and Tables

**Table 1:** Baseline demographic, clinical, and preoperative characteristics of patients undergoing radical cystectomy for urothelial bladder cancer, stratified by gender. Values are reported as median (interquartile range) for continuous variables and as number (percentage) for categorical variables. *p*-values are calculated using the Mann–Whitney U test for continuous variables and Chi-squared or Fisher’s exact test for categorical variables.

	Male	Female	*p*-Value
Age Diagnosis	68.5 (IQR = 13.8)	69.6 (IQR = 12.7)	0.83
Age Cystectomy	69.8 (IQR = 13.3)	70.5 (IQR = 12.6)	0.70
Clinical stage			0.77
cT1	208 (32.6%)	85 (36%)	
cT2	312 (48.8%)	121 (51.3%)	
cT3	69 (10.8%)	22 (9.3%)	
cT4	18 (2.8%)	5 (2.1%)	
Tis	23 (3.6%)	9 (3.8%)	
BMI	27.1 (IQR = 6.48)	26.7 (IQR = 6.58)	0.80
eGFR Createnine	71 (IQR = 28) 103.4 (IQR = 26)	73 (IQR = 25) 93.1 (IQR = 22)	0.03 0.01
Hb	126.8 (IQR = 25)	129.7 (IQR = 25)	0.21
Ethnicity			
White caucasian	459 (83.3%)	180 (84.9%)	0.81
Asian	17 (3.1%)	3 (1.4%)	
Black	30 (5.4%)	9 (4.2%)	
Mixed	3 (0.5%)	2 (0.9%)	
Other/prefer not to say	42 (7.6%)	18 (8.5%)	
Total			0.61
DM	75 (11.7%)	26 (10.6%)	0.72
CKD	41 (6.4%)	10 (4.08%)	0.24
Neoadjuvant Therapy	105 (21.6%)	33 (17.0%)	0.21
CIS present	222 (34.1%)	84 (34.3%)	0.96

BMI—body mass index, eGFR—estimated glomerular filtration rate; Hb—haemoglobin; DM—diabetes mellitus; CKD—chronic kidney disease; CIS—carcinoma in situ.

**Table 2:** Intraoperative variables of patients undergoing radical cystectomy for urothelial bladder cancer, stratified by gender. Values are reported as median (interquartile range) for continuous variables and as number (percentage) for categorical variables. *p*-values are calculated using the Mann–Whitney U test for continuous variables and Chi-squared test for categorical variables.

	Male	Female	*p*-Value
Total time of surgery	330 (IQR = 120)	330 (IQR = 90)	0.32
EBL	400 (IQR = 312.5)	400 (IQR = 425)	0.68
LN dissected	15 (IQR = 9)	16 (IQR = 12)	0.21
Technique			
ORC	194 (30.4%)	68 (27.8%)	
RARC	429 (67.1%)	172 (70.2%)	
Total			0.41
Complications (Clavien–Dindo ≥ 3)	58 (9.1%)	27 (10.9%)	0.07

EBL—estimated blood loss; ORC—open radical nephrectomy; RACR—robot assisted radical cystectomy.

**Table 3:** Pathological variables of patients undergoing radical cystectomy for urothelial bladder cancer, stratified by gender. Values are reported as number (percentage). *p*-values are calculated using the Chi-squared test or Fisher’s exact test, as appropriate.

	Male	Female	*p*-Value
pT stage			0.479
pT1	77 (12.5%)	34 (14.2%)	
pT2	138 (22.3%)	65 (27.2%)	
pT3	169 (27.3%)	64 (26.8%)	
pT4	43 (7%)	15 (6.3%)	
Tis *	90 (14.6%)	23 (9.6%)	
Variant Histology (at least 10%)			0.73
	87 (15.9%)	36 (16.9%)	
Adenocarcinoma	6 (6.9%)	2 (5.6%)	
Squamous	42 (48.3%)	16 (27.6%)	
Micropapillary	8 (9.2%)	5 (13.9%)	
Sarcomatoid	11 (12.6%)	3 (8.3%)	
Glandular	9 (10.3%)	5 (13.9%)	
Plasmacytoid	4 (4.6%)	1 (2.8%)	
Small cell	7 (8%)	4 (11.1%)	
			
PSM	41 (6.4%)	17 (6.1%)	0.87
Ureteric Invasion	14	4	
Urethral Invasion	15	6	0.67
Soft tissue ^	11	7	0.05

* present in final histology; ^ all with ≥pT3 on final histology specimen; PSM—positive surgical margins.

## Data Availability

The data presented in this study are available upon request from the corresponding authors.
